# Ultrafast Faraday rotation probe of chiral phonon-polaritons in LiNbO_3_

**DOI:** 10.1126/sciadv.aec8970

**Published:** 2026-09-04

**Authors:** Megan F. Biggs, Sin-hang (Enoch) Ho, Aldair Alejandro, Matthew J. Lutz, Clayton D. Moss, Jeremy A. Johnson

**Affiliations:** Department of Chemistry and Biochemistry, Brigham Young University, Provo, UT 84602, USA.

## Abstract

A strong magnetic response in materials after time reversal symmetry breaking excitation of circular ionic motion may open avenues for ultrafast control. By combining a pair of perpendicularly polarized terahertz pulses with the right relative delay, we create a chiral terahertz driving field to excite chiral phonon-polaritons in LiNbO_3_. The magnitude of the ultrafast Faraday rotation probe matches what we would expect from an internal magnetization of ∼10 $\mu_{N}$ per unit cell, which would require an external magnetic field in excess of 10 Tesla to produce. The Faraday rotation signal switches direction when the input terahertz pulse is changed from left- to right-circular polarization, indicating a change in magnetization direction. In addition, we improve upon previous experiments by using a differential chopping scheme to remove signals arising from linearly polarized terahertz components that can contaminate the Faraday signal. Models show that chiral atomic motion combines with the inverse Faraday effect to induce a magnetic moment in nonmagnetic LiNbO_3_.

## INTRODUCTION

Motion of charged particles can induce a magnetic field as described by one of Maxwell’s fundamental equations: $\nabla \times B=\mu \left(J+\epsilon_{0}\frac{\partial E}{\partial t}\right)$. The Ampere-Maxwell law states that a charged particle moving in a linear path will create a magnetic field perpendicular to the motion. That field can be amplified if the charged particles have angular momentum and move in a circular path (rather than linear) where the magnetic fields that are perpendicular to the plane of motion add together. There are many ways to cause charged particles to move in a circular path, such as simply applying electrical current through curled wires in a solenoid (*1*). Another method is to use circularly polarized light to drive the circular motion of charged particles. A well-known example of this magneto-optical phenomenon is the inverse Faraday effect (IFE), where circularly polarized light excites the electrons in a material to move in a circular path, and thus generate a transient magnetization when the driving light is present (*2*–*6*). An additional demonstration of this magneto-optical phenomenon is the phonon-magnetic effect (*7*–*11*): When circularly polarized light is directed into a material to drive ionic motion, the ions inside can gain angular momentum and rotate in a unidirectional path (so-called ion loops). In the most straightforward case, the ion loops are comprised of a pair of degenerate vibrational modes with orthogonal motion (E-symmetry modes in the material we study here). To achieve circular motion, the pair of degenerate modes must be excited with the correct relative phase delay. At times these excited ion loops have been termed “chiral phonons,” however chiral excitations include a third dimension or propagation direction that is lacking in simple two-dimensional (2D) motion (*12*). Chiral phonons naturally break time reversal symmetry (TRS), allowing materials to exhibit unique quantum phenomena, such as the quantum Hall effect, superconductivity, and magneto-electric effects as mentioned above (*10*, *13*–*15*).

This property of TRS breaking makes chiral phonons extremely useful in many novel applications, such as superconductors (*16*), spintronics (*17*), topological materials (*18*), and, the focus of this paper, magnetism (*19*–*21*). Achieving control of magnetic properties has great potential in data storage for electronic devices as a means to create ultrafast nonvolatile switches (*22*). Here, we demonstrate the detection of an ultrafast Faraday rotation signal through the TRS breaking excitation of chiral phonon-polaritons in LiNbO_3_, inducing a magnetization that turns on faster than any switch used in electromagnetic devices today.

Phonon magnetism via the excitation of ion loops has been experimentally observed in two recent publications. Luo *et al.* (*10*) induced a Faraday rotation of ultrafast probe pulses in paramagnetic CeF_3_ through phonon-spin coupling driven by circularly polarized terahertz (THz) light pulses. Basini *et al.* (*11*) created a phonon-induced magnetooptical Kerr rotation of ultrafast probe pulses in diamagnetic SrTiO_3_ (STO). STO has no innate magnetic ordering, meaning that they were able to switch on a magnetic-field driven Kerr signal in a non-magnetic material. Basini *et al.* (*11*) measured a Kerr rotation and reported a magnetization corresponding to an effective magnetic field four orders of magnitude larger than had been theoretically calculated (*7*, *9*, *23*). Whether or not the optical signals actually indicate such a strong magnetic field is under debate. Recently, Merlin (*24*) theorized that the magnetic fields induced by both Luo *et al.* (*10*) and Basini *et al.* (*11*) were non-Maxwellian fields due to the breaking of TRS caused by the circularly polarized THz, but not true magnetic fields. In this work, we share evidence that effects other than an internal magnetization can result in an ultrafast polarization rotation that may complicate optical detection of an induced magnetic response. However, we also report an isolated Faraday rotation with magnitude and dynamics in line with theoretical predictions.

The magneto-optic Faraday effect is traditionally observed by the rotation of linearly polarized laser light in the presence of an external magnetic field that induced an internal magnetization oriented in the light propagation direction. Here, we experimentally observe the creation of a strong Faraday rotation resulting from phonon-magnetic excitation with circularly polarized THz pulses, similar to (*10*, *11*). However, our sample and experimentation differ from these previous works in important ways: First, the LiNbO_3_ sample is a ferroelectric material where E-symmetry modes couple with light to form phonon-polaritons that propagate into the depth of the material (*25*–*27*), creating truly chiral ionic motion. Second, we can experimentally isolate polarization rotation signals that arise from linear polarization components of the chiral THz pump fields and remove these contributions from the measured response, in a way not possible in previous measurements. After these additional signal contributions are removed, we report a Faraday rotation corresponding to an induced internal magnetization of ∼10 $\mu_{N}$ that is roughly one order of magnitude larger than theoretically calculated for these modes in LiNbO_3_ (*9*).

Below we describe how we form chiral THz pulses to excite chiral phonon-polaritons in LiNbO_3_. We show how to isolate the ultrafast Faraday rotation signal due to chiral excitation and separate it from polarization rotation signal arising from linearly polarized THz pulses, model the signal contributions coming from both the IFE and the phonomagnetic effect, and show that this ultrafast Faraday rotation in originally nonmagnetic, ferroelectric LiNbO_3_ is commensurate with the polarization rotation that would arise from a ∼10 $\mu_{N}$ per unit cell magnetization, that in a traditional Faraday rotation measurement would require a >10 T external magnetic field. We also show that our measured response matches a modeled response, only if we include the phonon-magnetic response expected from the excited chiral phonon-polariton.

## RESULTS

### Exciting chiral phonon-polaritons

**Fig. 1.**
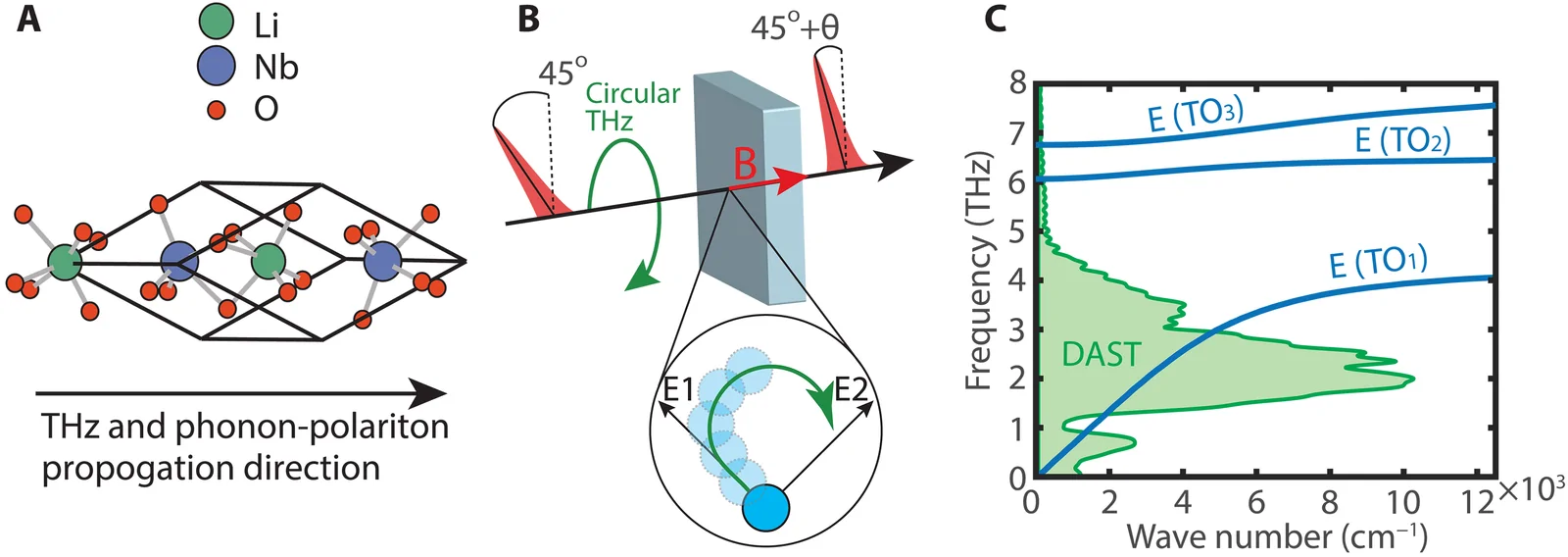
LiNbO_3_ unit cell, simple experimental diagram, and dispersion curve. (**A**) Unit cell of LiNbO_3_ with lithium atoms in green, niobium atoms in purple, and oxygen atoms in red. THz propagates along the *z* [001] axis. (**B**) Simplified experimental setup showing the probe (red) and circular THz pump (green), leading to the circular motion of the ions (blue circles), and an induced Faraday rotation θ. E1 and E2 indicate the axes of the degenerate phonon-polaritons. (**C**) Phonon-polariton dispersion curve of LiNbO_3_. Green shading shows a representative spectrum from the DAST THz generation crystal used in these experiments to excite along the lowest of three phonon-polariton E mode branches shown by blue lines [dispersion curve extracted from (*26*)].

We used DAST THz generation crystals (*28*) to generate two broadband THz pulses with frequency content ranging from 0.5 to 5 THz (THz-1 and THz-2, respectively). THz-1 was vertically polarized and THz-2 was horizontally polarized. The perpendicularly polarized THz pulses selectively excite the degenerate E (TO_1_) modes in LiNbO_3_ (unit cell of LiNbO_3_ shown in Fig. 1A). By changing the relative timing between the THz pulses using a delay stage in the THz-2 beam path, we controlled the ellipticity of the combined THz light that irradiates the sample (*29*) (detailed description of the experimental setup is in Supplementary Text).

In contrast to excitation of zone-center phonons with zero group velocity, broadband THz pulses can excite phonon-polaritons in LiNbO_3_ with frequencies at least up to ∼4 THz along the dispersion curve. Figure 1C shows the lowest three E-phonon-polariton branches in LiNbO_3_ (blue lines), where the lowest branch is excited by the THz spectrum (shaded green area) (*26*). With broadband circularly polarized THz pulses incident on a *z*-cut LiNbO_3_, we directly excite the phonon-polariton branches in both *x*- and *y*-crystallographic directions, with a relative delay to drive chiral ionic motions propagating into the material with the main spectral amplitude spanning 0 to 4 THz.

We spatially overlapped a focused 100-fs, 800-nm probe beam with the THz on the LiNbO_3_ sample (see Fig. 1B), measuring a change in polarization of the linear probe beam through balanced detection with dual photodiodes. We used a probe delay stage to capture a time window of ∼4 ps in a series of repeated scans. For each scan, to create different THz ellipticity angles, the relative delay between THz pulses was varied by step sizes equivalent to a 6.67 fs delay on the THz-2 delay stage.

### Isolating the Faraday signal from the linear sample response

In addition to selective excitation of the E(TO_1_) branches to produce a chiral phonon-polariton that induces an internal magnetization, we also isolate the Faraday rotation from signal contributions due to the linear polarization components of the chiral THz pump field. Multiple processes exist that can rotate the polarization of an ultrafast probe as is measured in Kerr or Faraday rotation measurements. For example, the probe polarization can change linearly due to interaction with an excited Raman-active vibration (*30*–*32*) or due to the electro-optic effect. The previous experiments of Luo (*10*) and Basini (*11*) do not isolate the Faraday or Kerr signal from this linear pump-induced probe rotation. A differential chopping scheme (*32*–*35*) allows us to isolate the Faraday signal from any probe rotations induced by linear components of the chiral THz excitation field.

A combination of optical choppers gives, for a sequence of four 800-nm probe pulses, the following pattern: both THz pulses, THz-1 only, THz-2 only, and no THz pulses. To isolate the chiral signal, we subtract the THz-1 and THz-2 pulse responses from the response containing both THz pulses, each with the probe signal in the absence of THz removed (see Eq. 1).

$$S_{\text{chiral}}=\left(S_{\text{both}}-S_{\text{none}}\right)-\left(S_{\text{THz}-1}-S_{\text{none}}\right)-\left(S_{\text{THz}-2}-S_{\text{none}}\right)$$ (1)

This chiral signal isolation allows us to eliminate artifacts arising from the linearly polarized components of the chiral THz pump. $S_{\text{chiral}}$, as detected in the polarization sensitive Faraday rotation scheme described in Fig. 1, measures the induced magnetic moment (oriented in the same or opposite direction to the probe propagation) as a function of time. Because of the Faraday effect, the polarization of a linearly polarized incident probe is rotated clockwise or counterclockwise around the direction of probe propagation when it passes through a medium influenced by a magnetic field. The magnitude of the polarization rotation in a traditional Faraday rotation measurement depends linearly on an external magnetic field (*B*), the material-dependent Verdet constant (v), and the thickness (*L*) of the medium, as shown in Eq. 2. In addition, with the vacuum permeability ($\mu_{0}$) and the volume magnetic susceptibility ($\chi_{V}$), we see alternatively how an induced internal magnetization (*M*) leads to the Faraday rotation

$$\Delta \theta =\mathrm{vBL}=v\;\mu_{0}\frac{1}{\chi_{V}}\text{M L}$$ (2)

**Fig. 2.**
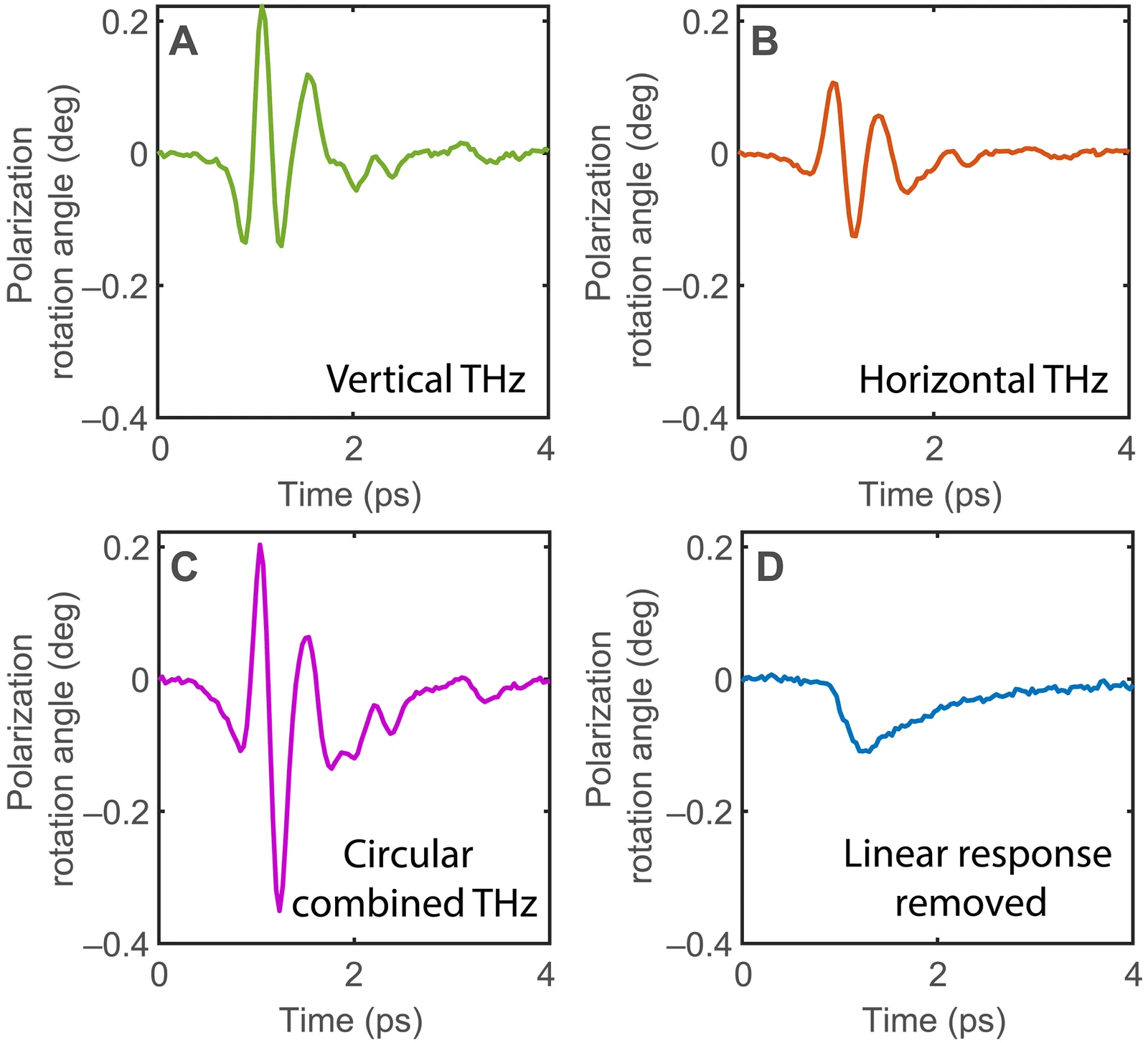
Four signals isolated with the differential chopping scheme from measurements with two perpendicularly polarized THz pump pulses. (**A** and **B**) Signals from the vertical THz-1 pump and the horizontal THz-2 pump, respectively. (**C**) Signal when both THz pulses are present to produce a chiral THz excitation pulse, showing clear contamination due to linear field response shown in (A) and (B). (**D**) Isolated Faraday rotation signal obtained from (C) after removing the linear field components shown in (A) and (B).

Figure 2 shows signal recorded with four pulse sequences that are separated with differential chopping. Figure 2A shows the measured signal when only the vertically polarized THz-1 pulse excites the sample; the probe polarization is rotated by a linear (not circular) excitation, which will not induce a TRS breaking magnetic-field driven Faraday signal. Presumably, these signals arise from the 800-nm probe interacting with the linearly polarized E phonon-polariton through a Raman process, which rotates the polarization of scattered probe light (*25*, *30*). Figure 2B shows a similar polarization rotation induced by the phonon-polariton motion driven by the linearly polarized horizontal THz-2 pump. Figure 2C shows the sample response when the combined circularly polarized THz pulse excites the sample, where we can see that the signals from the linearly polarized V and H field components are present in the signal from the circularly polarized THz pulse. This combined signal in Fig. 2C shows artifacts from the linear components of the circular THz pump and is equivalent to the signals reported by Luo *et al.* (*10*) and Basini *et al.* (*11*).

As mentioned above, a differential chopping scheme can remove these artifact signals from the combined LiNbO_3_ response by subtracting the linear pump signals shown in Fig. 2 (A and B) from the combined signal in Fig. 2C to isolate the Faraday rotation signal that arises purely due to the circularly polarized THz pump (Fig. 2D). The shape of the isolated chiral signal is markedly different than the shape of the other three isolated pulses because it arises purely from the chiral phonon-polariton excitation. However, the fact that a linearly polarized pump can induce a probe polarization rotation suggests that we must be careful in ascribing even our pure chiral pump signal solely to a magnetic Faraday response. In the following, we discuss the chiral signal as being a signature of an induced magnetization in the material, and below, we revisit other possibilities.

### Faraday signal inverts with handedness of circularly polarized THz light

**Fig. 3.**
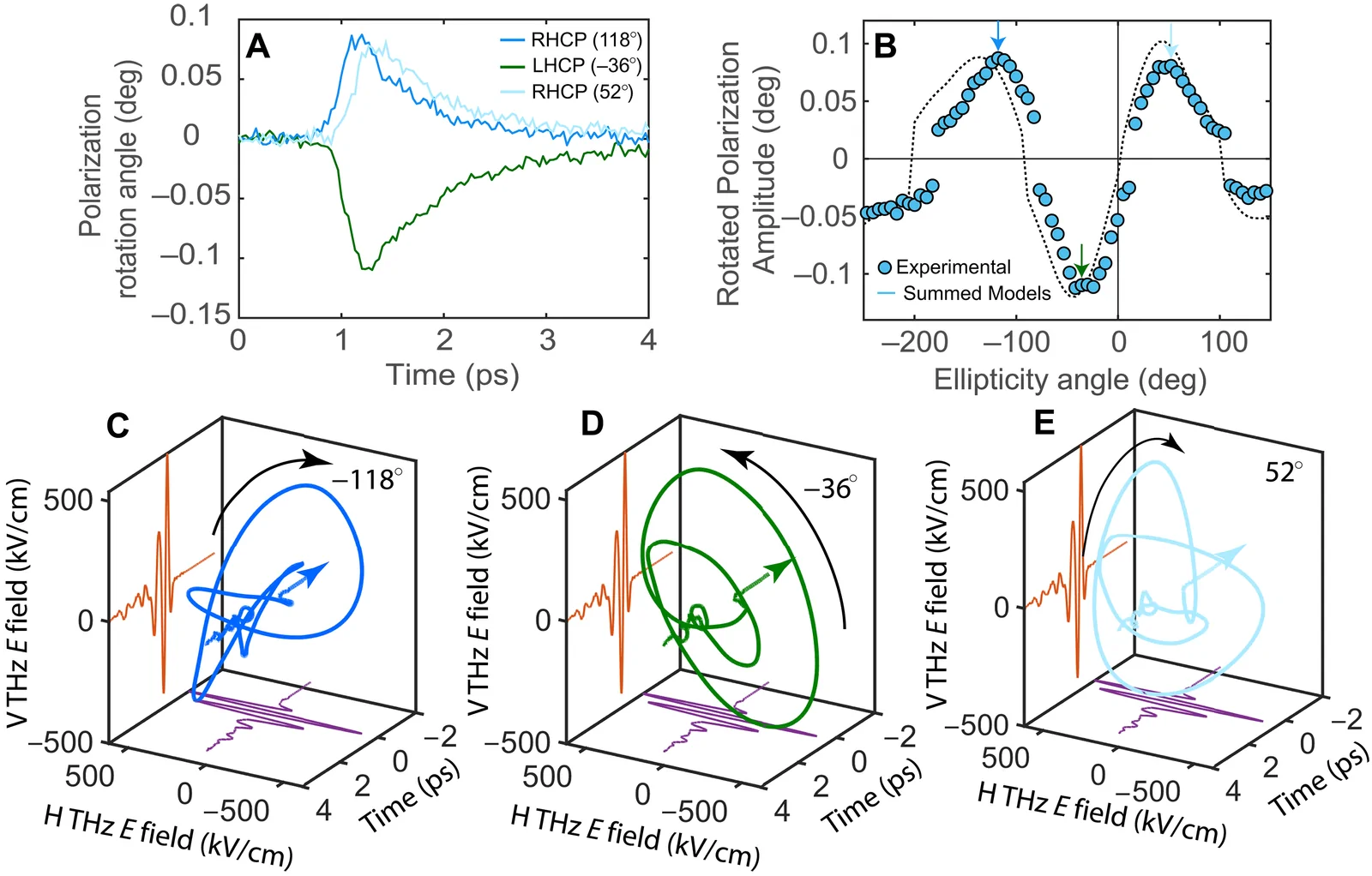
Faraday rotation signals in response to varying ellipticities of THz input pulse. (**A**) Isolated Faraday nonlinear signal arising from rotation of the probe polarization at the three most circular ellipticity angles. Green is the response from LHCP input THz light and the blues are the response from RHCP input THz light. (**B**) Faraday rotation signal magnitude as a function of ellipticity angle. The arrows indicate the delays plotted in (A). The modeled signal is shown in black. (**C** to **E**) Combined field of the THz pulses along with their horizontal and vertical components at each of the resulting ellipticity angles plotted in (A).

**Fig. 4.**
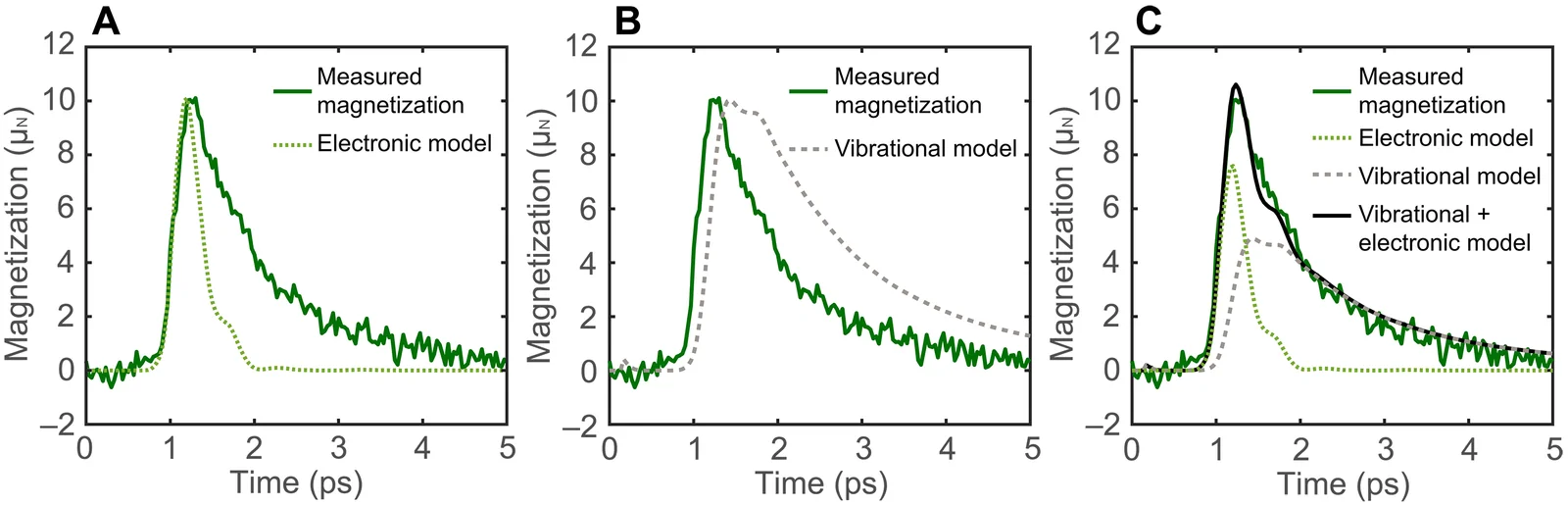
Modeled electronic and vibrational magnetic moment contributions. Experimental data of the induced magnetization per unit cell (units of nuclear magneton $\mu_{N}$) compared to the modeled magnetization from (**A**) the electronic (IFE) contribution, (**B**) phonon-magnetic contribution, and (**C**) the linear combination of both contributions. The *y*-axis magnitude is determined using the measured probe polarization rotation, together with the Verdet constant and magnetic susceptibility for LiNbO_3_ according to Eq. 2.

In Fig. 3A, we show the chiral-pump driven Faraday rotation at the three most circular ellipticity angles of the THz pump: −118° (Fig. 4C), −36° (Fig. 4D), and 52° (Fig. 4E) (see the Supplementary Materials for how the “average” ellipticity angle for these broadband THz pulses was determined).

Figure 3A shows the isolated Faraday rotation arises on an ultrafast, subpicosecond timescale. The Faraday rotation changes sign when switching from right-handed circularly polarized (RHCP) THz to LHCP input THz light, as expected for a signal arising from the IFE and the circular motion of ions. (Note that we define handedness from the view behind the THz pulse, looking in the direction of propagation). The change from right- to left-handed polarization reverses the order that the two phonon-polariton E modes are excited, changing the direction of ionic rotation, and reversing the direction of an induced magnetic field. In Fig. 3B, we plot the Faraday rotation signal amplitude against the ellipticity angles and compare to a model of the ellipticity angle signal dependence (discussed in more detail below). This shows the magnitude of the Faraday signal changes with ellipticity angle, once again noting that the highest signals occur when the input THz pulse is more circular and essentially zero signal when the THz polarization and induced ionic motion is linear (0° ellipticity angle).

## DISCUSSION

### Separating phonon-magnetism from the IFE

The isolated Faraday rotation signal follows the expected ellipticity angle dependence due to an induced magnetization. However, in addition to the phonon-magnetic effect, an induced magnetic moment could also result from the inverse Faraday effect (IFE), where the circular motions of electrons in a material driven directly by the THz electric field induce a magnetization (*5*). To determine whether a vibrational or electronic mechanism was creating the magnetization that leads to the Faraday rotation in LiNbO_3_, we mathematically modeled the response from both processes.

Based on Juraschek and coworkers’ work (*7*), the magnetic moment of a charged particle is proportional to its angular momentum, and the angular momentum can be calculated from the motion of the charged particle as shown in Eq. 3. In Eq. 3, M is the induced magnetization, γ is a gyromagnetic ratio, and L is the angular momentum, while Q and $\dot{Q}$ are the motion and velocity the charged species, respectively.

$$M=\gamma L=\gamma Q\times \dot{Q}$$ (3)

For the phononic contribution, for Q, we use $Q_{i}$, the normal coordinate of the phonon mode motion. We model $Q_{i}$ by computing the total motion of the phonon-polariton when excited with a broadband THz pulse by solving the differential equation of motion Eq. 4 for each point on the dispersion curve (described in more detail in the Supplementary Materials).

$${\ddot{Q}}_{i}+2\Gamma_{i}{\dot{Q}}_{i}+\omega_{i}^{2}Q_{i}=Z_{i}^{*}E$$ (4)

In Eq. 4 (*32*, *36*, *37*), $Z_{i}^{*}$ is the mode effective charge, $Q_{i}$ represents the vibrational coordinate, i represents a particular point on the dispersion curve, with $\Gamma_{i}$ the damping rate and $\omega_{i}$ the angular frequency for that point. Ε is the THz driving field. We input the $\Gamma_{i}$ and $\omega_{i}$ values from (*25*) to calculate a separate $Q_{i}$ for each point on the dispersion curve. The Ε input is the experimentally measured time trace of the THz pulse generated with DAST. By modeling in this manner, we generate an output of the vibrational mode motion ($Q_{i}$) at each frequency ($\omega_{i}$) that is excited by the THz bandwidth. We perform these calculations twice: once with the vertically polarized THz-1 field and once with the variably delayed horizontally polarized THz-2 field, which gives full phonon-polariton motion for each relative pump delay. We calculate an induced magnetization using Eq. 3 for each point along the phonon-dispersion curve and then sum up all the individual magnetic contributions to obtain a total magnetization for the sample, and we increased the density of points along the dispersion curve until no additional change is observed. Similarly, the electronic contribution is also in the form of Eq. 3; however, now we need to consider the angular momentum of electrons instead, driven nonresonantly by the THz electric field $E_{i}$. Replacing Q in Eq. 3 with the THz electric field vector enables us to model the IFE response ($M\propto E_{i}\times {\dot{E}}_{i}$) as well (*7*). To accurately account for the signal, we mathematically convolved the signal with the probe pulse, which has a pulse duration of 100 fs.

In Fig. 4, we plot the Faraday rotation signal, converted to internal magnetization per unit cell, for the THz ellipticity angle (−34°) that gives the largest magnitude Faraday rotation of ∼−0.1°. The polarization rotation magnitude is in line with what would result from an external magnetic field strength in excess of 10 T, calculated using a Verdet constant of 0.2°/T per cm from (*38*). We confirmed this value for the Verdet constant as described in the Supplementary Materials. There is no 10-T magnetic field induced in these measurements, but in diamagnetic LiNbO_3_, such an external field would induce an internal magnetization of ∼10 $\mu_{N}$ per unit cell (determined using the magnetic susceptibility of LiNbO_3_ of $\chi_{V}=-5.51\times 10^{-5}$, measured at room temperature with a vibrating magnetometer). Therefore, we correlate the observed Faraday rotation with an internal magnetization reaching ∼10 $\mu_{N}$ per unit cell.

Figure 4 shows that neither pure electronic (IFE) (Fig. 4A) nor pure phonon-magnetic (Fig. 4B) modeled contributions alone match the observed signal. In principle, both contributions can be present, so we form a linear combination of the two models where the only free parameter is the relative magnitude of each (black in Fig. 4C). This linear combination agrees very well with the experimental data, suggesting that both phononic and electronic contributions lead to the TRS-breaking Faraday signal. Using the scaling factor from the fit at the −34° ellipticity angle, we show this combined model is applicable, and we see good agreement with the data for every THz ellipticity angle, plotted as the dashed line in Fig. 3B above.

The magnitude of the induced magnetization we observe is similar to what has been theoretically calculated for LiNbO_3_ (*9*). If we scale the results from (*9*) appropriately to our experimental THz field strengths, then they predict an internal magnetization of ∼0.5 $\mu_{N}$ per unit cell, for a rhombohedral unit cell. Our modeling and fit to the data, including the magnetic susceptibility of LiNbO_3_, suggests a similar phonon-magnetic contribution to our observed signal of ∼5 $\mu_{N}$ per unit cell—only one order of magnitude larger. We note that in (*9*), the phonon-polariton nature of these degenerate E(TO_1_) modes in LiNbO_3_ was neglected, leading to an underestimate of the induced polarization and induced magnetization upon chiral THz excitation that would explain our larger value.

In conclusion, we have excited ultrafast TRS-breaking chiral motion of phonon-polaritons, resulting in an ultrafast Faraday signal that matches the probe polarization rotation expected from a >10 Tesla external magnetic field. This large magnetic field strength is based on the Verdet constant measured with an applied external magnetic field. Thus, we emphasize that the magnitude of the Faraday rotation signal we observe matches the Faraday rotation one would expect for an induced internal magnetization of ∼10 $\mu_{N}$ per unit cell that is in line with theoretical predictions of phonon-magnetism in LiNbO_3_.

Because of some remaining uncertainty in the field, we also close with a proposal: To differentiate the cause of the observed strong Faraday rotation and better understand the strength and origin of an internal magnetization and induced magnetic field, experiments must be performed with a variety of probes that will be sensitive to the induced magnetic moment on ultrafast timescales. This may include x-ray probes of magnetic order, Zeeman splitting of appropriate states in adjacent materials, internal dopants that would be influenced by the induced magnetization, or experiments showing the modification of inherent internal magnetic order in an excited material due to a phonon-magnetic excitation. These measurements, combined with additional theoretical work, are needed to support or require us to rethink the optical probe measurements to date.

## MATERIALS AND METHODS

We used the 2D THz experimental setup shown in fig. S1 and described in (*29*) and (*32*). The (1450 nm) and idler (1790 nm) outputs from an optical parametric amplifier were directed into separate DAST THz generation crystals to generate broadband THz pulses with frequency content ranging from 0.5 to 5 THz (THz-1 and THz-2, respectively). THz-1 was vertically polarized (peak-to-peak field strength of 981 kV/cm), and THz-2 was horizontally polarized (peak-to-peak field strength of 1151 kV/cm), leading to the creation of perpendicularly polarized THz pulses to selectively excite the degenerate E modes in LiNbO_3_. The two THz pump pulses were directed into a three-parabolic mirror scheme and focused onto the same area of the 500-μm thick, *z*-cut LiNbO_3_ with a spot size of ∼250 μm (see fig. S1). By changing the relative timing between the THz pulses using the delay stage in the THz-2 beam path, the ellipticity of the combined THz light that irradiates the sample is controlled.

We spatially overlapped a focused 800-nm probe beam with the THz on the LiNbO_3_ sample. Using the first HWP, we rotated the probe beam to a polarization of 45° before striking the sample. The second HWP ensured the probe beam was oriented, such that an equal amount of vertically and horizontally polarized probe light struck the Wollaston prism, allowing compensation for any strain-induced birefringence in the sample and also allowing for rotating the initial probe polarization if needed. We measured a change in polarization of the linear probe beam through balanced detection with dual photodiodes. We varied the probe delay to record a series of scans over a probe range of ∼4 ps. For each of several scans, the relative delay between THz pulses was varied by step sizes equivalent to a 6.67 fs delay on the THz-2 delay stage, which allowed the measurement of the chiral signal at varying THz ellipticity angles.

## Supplementary Materials

### This PDF file includes:

Supplementary Text Table S1 Figs. S1 to S8 References
