# Supplementary material for: Ultrafast Faraday rotation probe of chiral phonon-polaritons in LiNbO3
Source: Sci Adv. 2026 Sep 4;12(36):eaec8970. doi: 10.1126/sciadv.aec8970 (PMC13544201; doi:10.1126/sciadv.aec8970)
Supplement: Supplementary file 1 [file sciadv.aec8970_sm.pdf]

Science Advances
AAAS

Supplementary Materials for

Ultrafast Faraday rotation probe of chiral phonon-polaritons in $LiNbO_3$

Megan F. Biggs *et al.*

Corresponding author: Jeremy A. Johnson, jjohnson@chem.byu.edu

Sci. Adv. **12**, eaec8970 (2026)
DOI: 10.1126/sciadv.aec8970

This PDF file includes:

Supplementary Text
Table S1
Figs. S1 to S8
References

Supplementary Text

S1. Phase Matching Considerations

$LiNbO_3$ belongs to the R3c space group, the character table and product table for that space group are shown below.

Table S1. Character table for R3c space group.

	E	$2C_3(z)$	$3\sigma_v$	linear, rotations	quadratic
A_1	1	1	1	z	x^2+y^2, z^2
A_2	1	1	-1	R_z	
E	2	-1	0	(x,y)(R_x,R_y)	(x^2-y^2,xy)(xz,yz)

The z-cut $LiNbO_3$ from this study has perpendicular x and y axes along in the sample plane that interacts with the terahertz pulses that propagate in the z-direction and polarized in the x-y plane. As shown in the character table, E modes lie along the x and y axes are both IR-active and Raman active. With perpendicularly polarized THz pulses (polarized in the x-y plane), both perpendicular E-phonon polaritons are coherently excited.

For an x-cut LiNbO3, the phase-matching equation for the phonon-polariton [see Refs (25,27,30,31)] is as follows for a phonon polariton traveling in the forward (+) direction:

$$k_+ = \frac{n_0}{c}\omega - \frac{n_e}{c}(\omega - \Omega_+),$$

where k_+ is the phonon polariton wavevector, and the two terms on the right are wavevectors for the scattered and incident probe light. For propagation in the x-direction, the probe experiences the extraordinary refractive index (n_e) when polarized in the z-direction, and when polarized in the y-direction experiences the ordinary refractive index (n_0). As seen in the equation, the difference in refractive indices for the incident and scattered probe light (which depend on probe wavelength) lead to specific wavevectors (k+) that we can be observed along the dispersion curve, that depend on the probe wavelength (25,27).

However, the current measurements are performed on a z-cut $LiNbO_3$ where the probe propagates in the z-direction and is polarized in the x-y plane. Therefore, the probe pulse (both incident and scattered light) will only experience the ordinary refractive index (n_o). That fact changes the phase-matching equations as follows:

$$k_+ = \frac{n_0}{c}\omega - \frac{n_0}{c}(\omega - \Omega_+)$$

Solving these equations for k_+ we get:

$$k_+ = \frac{n_0}{c}\Omega_+$$

This phase-matching equations suggests that no specific wavevector is observed, because this condition doesn't match the phonon dispersion curve. So the signal from a linearly polarized THz pulse alone, rather than coming from a specific point on the phonon-polariton dispersion curve, is essentially an electro-optic signal (see Figure 2A-B).

S2. Verdet Constant Measurements

We experimentally confirmed the Verdet constant from Ref. (*20*) through Faraday rotation measurements (see fig. S2). In these measurements, we send the horizontally polarized probe beam through a Wollaston prism, splitting the beam into a large horizontal and a small (~0) vertical component which we direct into two different photodiodes. To create a static magnetic field, we place a neodymium magnet (1.32 T) directly front of the $LiNbO_3$ sample. The magnet has a hole in the center, which allows the probe beam to pass through the magnet unfettered and then transmits through the $LiNbO_3$, followed by a Wollaston prism to split resulting vertical and horizontal components. We record the magnitude of light hitting both photodiodes in five scenarios: 1) probe beam blocked, 2) probe beam unblocked with no $LiNbO_3$ or magnet, 3) probe beam unblocked with only the $LiNbO_3$ in the path, 4) probe beam unblocked with only the magnet in the path, and 5) probe beam unblocked with both the magnet and $LiNbO_3$ in the path. We subtract the reference with the light blocked from the other measurements. The magnet alone measurements assure us that the magnet itself is not blocking any probe amplitude because we get the same signal level with and without the magnet in, if there is no $LiNbO_3$. We then calculate the Faraday rotation angle based on the difference between the $LiNbO_3$ alone measurements and the $LiNbO_3$ + magnet measurements.

To calculate the Verdet constant (ν) we use the typical Faraday rotation equation shown below (Eq. S1). We have rearranged the equation to solve for ν, where L is the thickness of the $LiNbO_3$ (500 µm), B is the strength of the magnetic field applied to the crystal (0.537 T, calculated based on our experimental setup and distance between the magnet and sample), and $\Delta\theta$ is the experimental change in polarization between the $LiNbO_3$ alone and the $LiNbO_3$ + magnet measurement. We only take into account the change in the small amount of vertically polarized light to calculate the small polarization rotation.

$$\nu = \frac{\Delta\theta}{BL} \qquad \text{Eq. S1}$$

We measured a Verdet constant comparable to the Verdet constant from Ref. (*20*).

S3. Modeling the Phonon-Polariton Motion

We modeled the total motion of the phonon-polariton when excited with a broadband THz pulse. We began by manually extracting the phonon-polariton dispersion curve and damping rates from Fig. 5 in Ref. (*25)*. For the dispersion curve, we performed a fit on the extracted values to create an equation for the curve (Eq. S2) (plotted in fig. S3). Using Eq. S2, we calculate frequencies for 500 evenly spaced points on the dispersion curve between 1000 cm^{-1} and 20000 cm^{-1}. The odd shape of the damping rate limited our ability to accurately fit it with an equation. Instead, we splined the extracted values from Ref. (*25*) and in this case created 500 data points evenly spaced between 0 cm^{-1} and 20000 cm^{-1} plotted in fig. S3.

$$\omega_i = 4\,\pi(-3.747 \times 10^{-17}x^4 + 2.937 \times 10^{-12}x^3 - 8.142 \times 10^{-8}x^2 + 9.723 \times 10^{-4}x - 0.1832) \quad \text{Eq. S2}$$

We then solve the differential equation of motion (Eq. S3) for each point on the dispersion curve.

$$\ddot{Q}_i + 2\Gamma_i\dot{Q}_i + \omega_i^2 Q_i = Z_i^* E \quad \text{Eq. S3}$$

In Eq. S3, Q_i represents the vibrational coordinate of a particular part of the dispersion curve i, Γ_i is the damping rate, ω_i is the angular frequency, Z_i^* is the mode effective charge, and E is the electric field. We input the Γ_i and ω_i values from fig. S3, and we calculate a separate $Q_i(t)$ for each of 500 discrete points along the dispersion curve. The THz electric field E used is an experimentally measured time trace of our THz pulse generated with DAST. $Z_i^*$was an arbitrary constant value kept the same for each point on the dispersion curve. This outputs a model of the vibrational mode motion at each frequency in arbitrary units. Fig. S4A and S4B show the vibrational motion and its Fourier transform at 4 different points along the dispersion curve near 1, 2, 3, and 4 THz. The damping rate is lower at low frequencies so the oscillations at the lower frequencies last longer in time, while the high frequency oscillations damp out quickly. The amplitude of the oscillations relates to the THz amplitude being input at the specific frequencies of the oscillations. We model the vibrational coordinate for both perpendicular branches of the phonon-dispersion curve through the same process, but change the experimental THz pulse that is read into the differential equation from the vertical THz-1 trace to the horizontal THz-2 trace.

The calculated Q_i values along the dispersion curve are then used to calculate the magnetic moment that arise from individual vibration as shown in Eq. 3. To simplify the process, we treat the gyromagnetic ratio as unity and look at the time-resolved shape of the response. The strength of the overall magnetic field is equal to the sum of M_i at all frequencies together for the entire phonon dispersion curve. We perform this calculation at every delay between the THz pulses. Fig. S5A shows the individual magnetic field models calculated at 4 frequencies along the phonon dispersion curve. Fig. S5B shows the totals sum of those 4 modeled magnetic moments. These are the modeled magnetic moments only at the delay between THz pulses where the largest Faraday signal was isolated (at an ellipticity angle of -34°). For the phonon-magnetic model presented in Fig. 4, instead of using only 4 points along the dispersion curve, we used 500 points along the dispersion curve. Adding additional points beyond 500 did not alter the modeled phonon-magnetic signal.

We modeled the magnetic field induced by electronic contribution (inverse Faraday effect) with using the measured THz electric field and the equation: $M \propto E_i \times \dot{E}_i$.

S4. Calculating the Ellipticity Angle

We calculate the ellipticity angle of the combination of the horizontal and vertical THz pulse at each delay between pulses. Determining true ellipticity angles for a few-cycle pulse is not exact (see Ref. (*39*)), so we calculate average ellipticity angle directly from the delay between the THz pulses. We chose to use DAST THz generation crystals for these experiments because the two-cycle THz pulses simplify determination of an ellipticity angle, compared to a more efficient THz generator like PNPA (*40*), which generates approximately single-cycle THz pulses.

Before every set of sample measurements, we use electro-optic sampling to measure the vertical and horizontal THz pulses at a single delay. In post, we shifted the initial horizontal pulse experimental measurement to the correct delay for each stage positions in the $LiNbO_3$ measurements based on the difference in stage position of this initial measurement and the stage positions of the subsequent measurements on the $LiNbO_3$ sample. From the subsequent shifted horizontal pulse scans and the initial vertical pulse scan, we calculate the ellipticity angle using three different methods, all based on Eq. S5.

$$\frac{\Delta x}{2\lambda} = \text{ellipticity angle} \qquad \text{Eq. S5}$$

Where λ is the wavelength of the horizontal THz pulse and Δx is the averaged distance between peaks in the horizontal and vertical THz pulses. We use three different methods to calculate λ, shown in fig. S6. For Method 1 (fig. S6A), we assume λ is the distance between the first two maxima. For Method 2 (fig. S6B), we calculate as twice the distance between the first large maxima and the largest minima. For Method 3 (fig. S6C), λ is determined as twice the distance between the largest minima and the second maxima.

We then calculate Δx at each stage position based on the delay between the horizontal and vertical THz pulses. We calculate a separate Δx for each major peak in the THz traces (shown in fig. S7) and then average the three calculated Δxs to get a single value for Δx to use in Eq. S5.

For the delay shown in fig. S7, which is also the delay in the main paper referred to as LHCP, these three methods were used and calculated ellipticity angles of -36°, -33°, and -31°. The ellipticity angle used in Fig. 4 were calculated with Method 2. Fig. S8 shows the phase difference between the vertical and horizontal THz pulses for the three ellipticity angles used in Fig. 4. We see for the THz pulse we label LHCP, the horizontal THz pulse arrives slightly before the vertical THz pulse. And for the two THz pulses we label RHCP, the vertical THz arrives slightly before the horizontal (for the first main oscillation at +52° and for the second main oscillation for -118°).

We note that the most circular polarization of the combined THz pulses should occur at 45° and -45°, so our calculation of the ellipticity angle is imperfect. This can likely be attributed to the fact that the two input THz pulses are generated using different DAST THz generation crystals, and therefore the shapes of the waveforms are not identical, affecting the ellipticity angle calculation. It may also be that the sample position in the focus of the EO crystal when measuring the THz pulses differs slightly from the $LiNbO_3$ position in the focus, slightly changing the delay between pulses and leading to a small offset between experiment and theory in Fig. 4B.

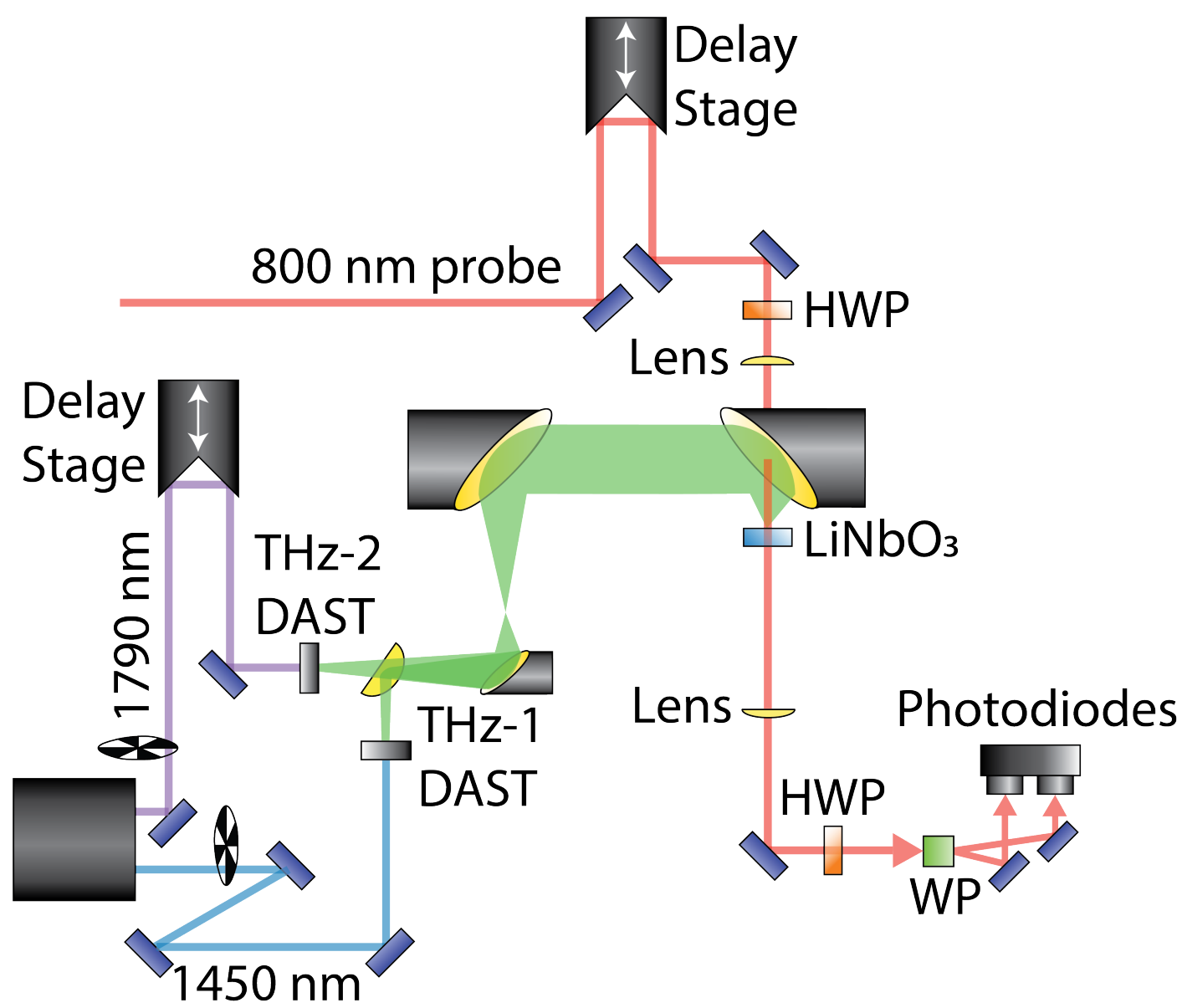

Fig. S1. 2D THz experimental setup for chiral excitation. A pair of perpendicularly polarized THz pump beams (green) were created using ultrafast pulses of IR-light (purple and blue) and then focused onto the sample using a series of three parabolic mirrors. Delay between the THz pulses can be changed using the delay stage in the IR-pump path to control the ellipticity of the combined THz that excites the sample. The 800-nm probe beam was also focused onto the sample. Half-wave plates (HWP) in the probe path control the linear polarization of the Faraday probe and ensure the probe is polarized at 45 degrees at the Wollaston polarizer (WP) (in the absence of a sample response). The WP separates horizontal and vertical probe components to measure the polarization rotation with balanced photodiodes as a function of time with respect to the THz excitation.

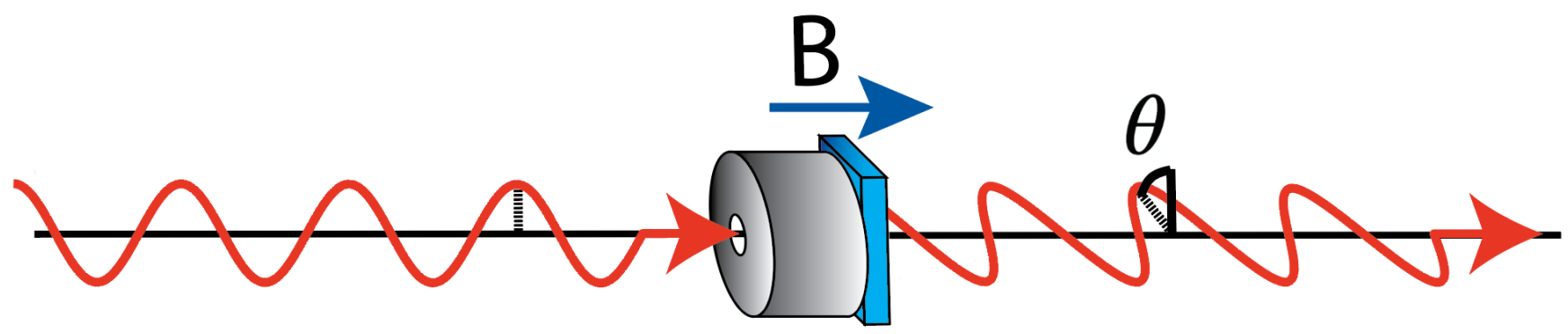

Fig. S2. Faraday rotation measurements of the Verdet constant of $LiNbO_3$. The linearly polarized probe (red) rotates through the Faraday effect in the presence of a static magnetic field (caused by the gray magnet), which is directed through a Wollaston prism and into two photodiodes.

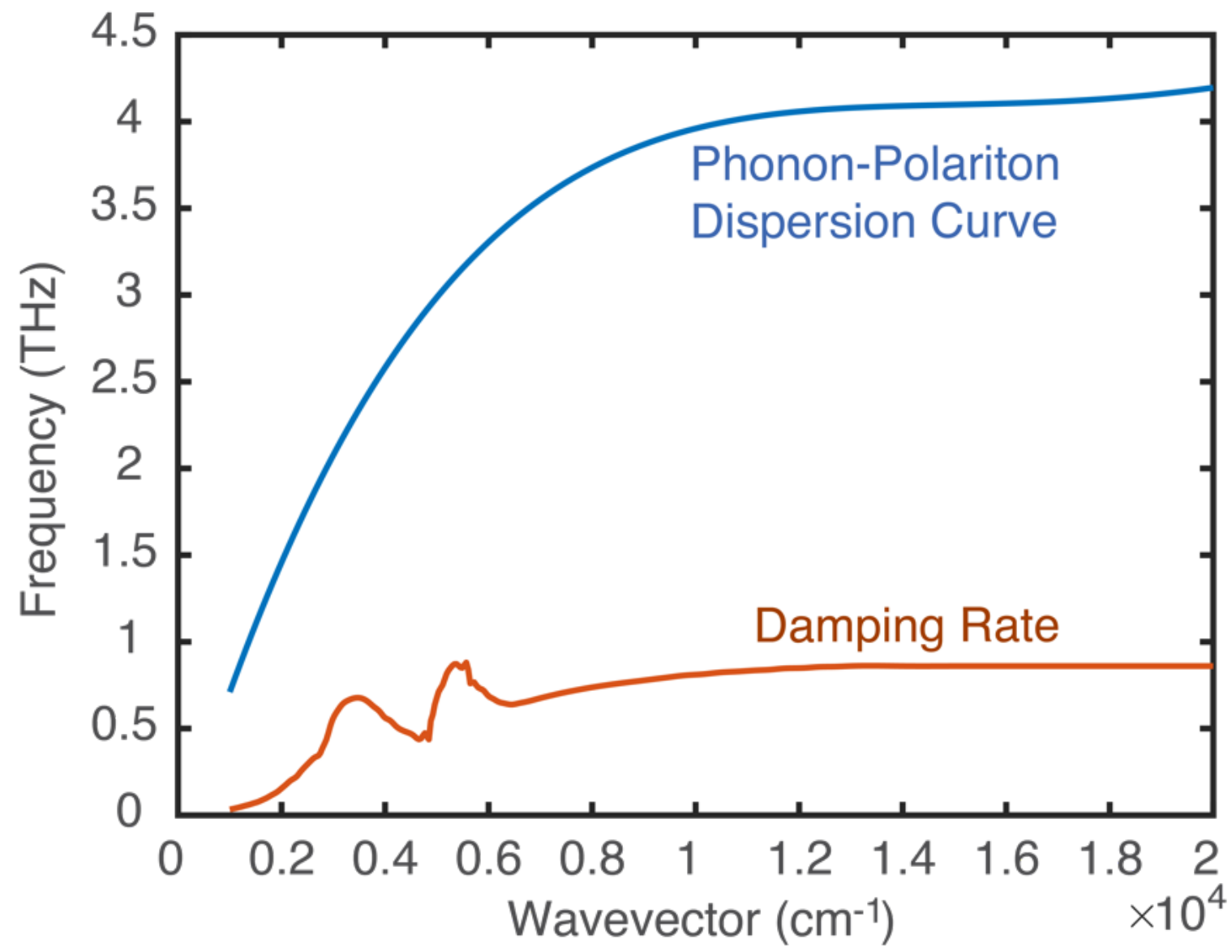

Fig. S3. **Damping rate and phonon-dispersion curve for $LiNbO_3$.** The E mode phonon-dispersion curve (blue) and damping rate (red) for $LiNbO_3$ extracted from Ref. (*25*).

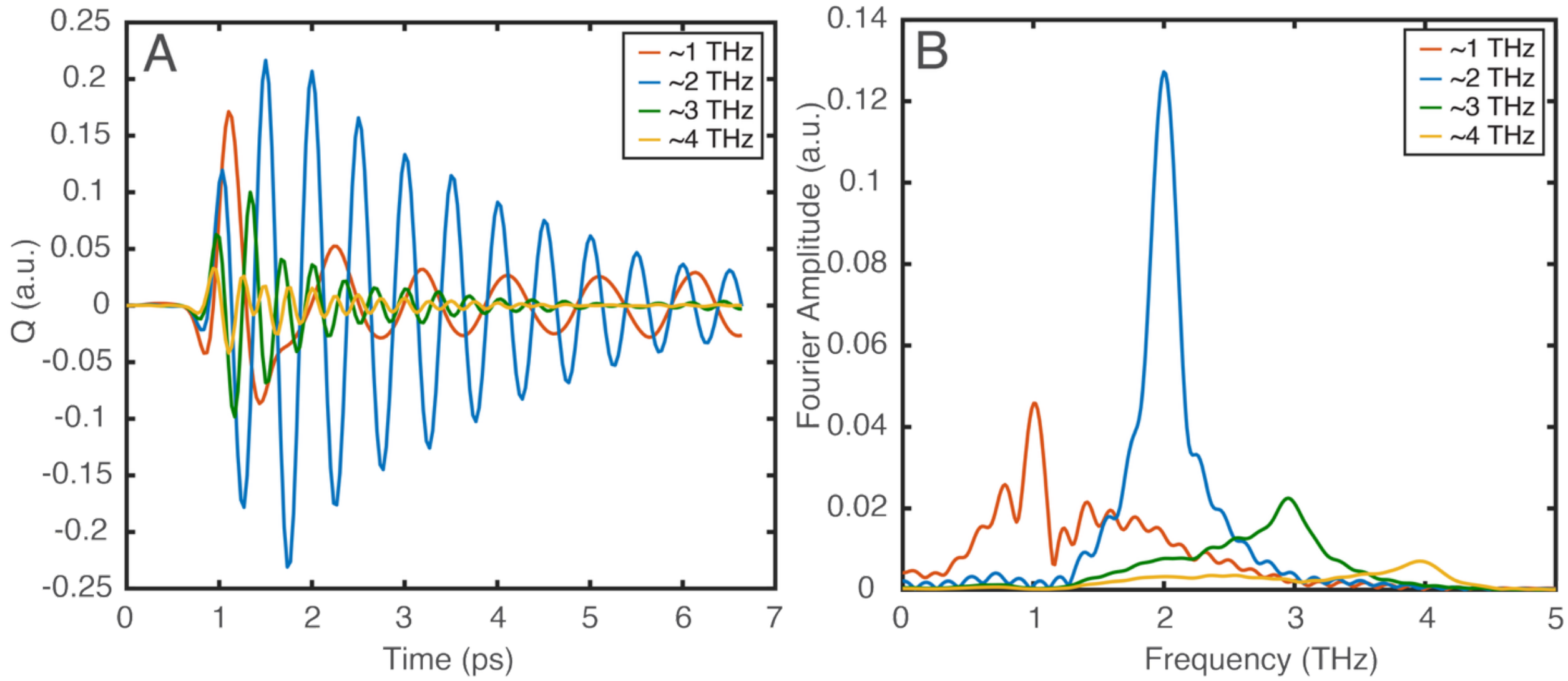

Fig. S4. Example modeled vibrational motion and frequency content. (**A**) The modeled vibrational coordinate of the E phonon-polariton at four frequencies on the dispersion curve and (**B**) the Fourier transform of those vibrational coordinates.

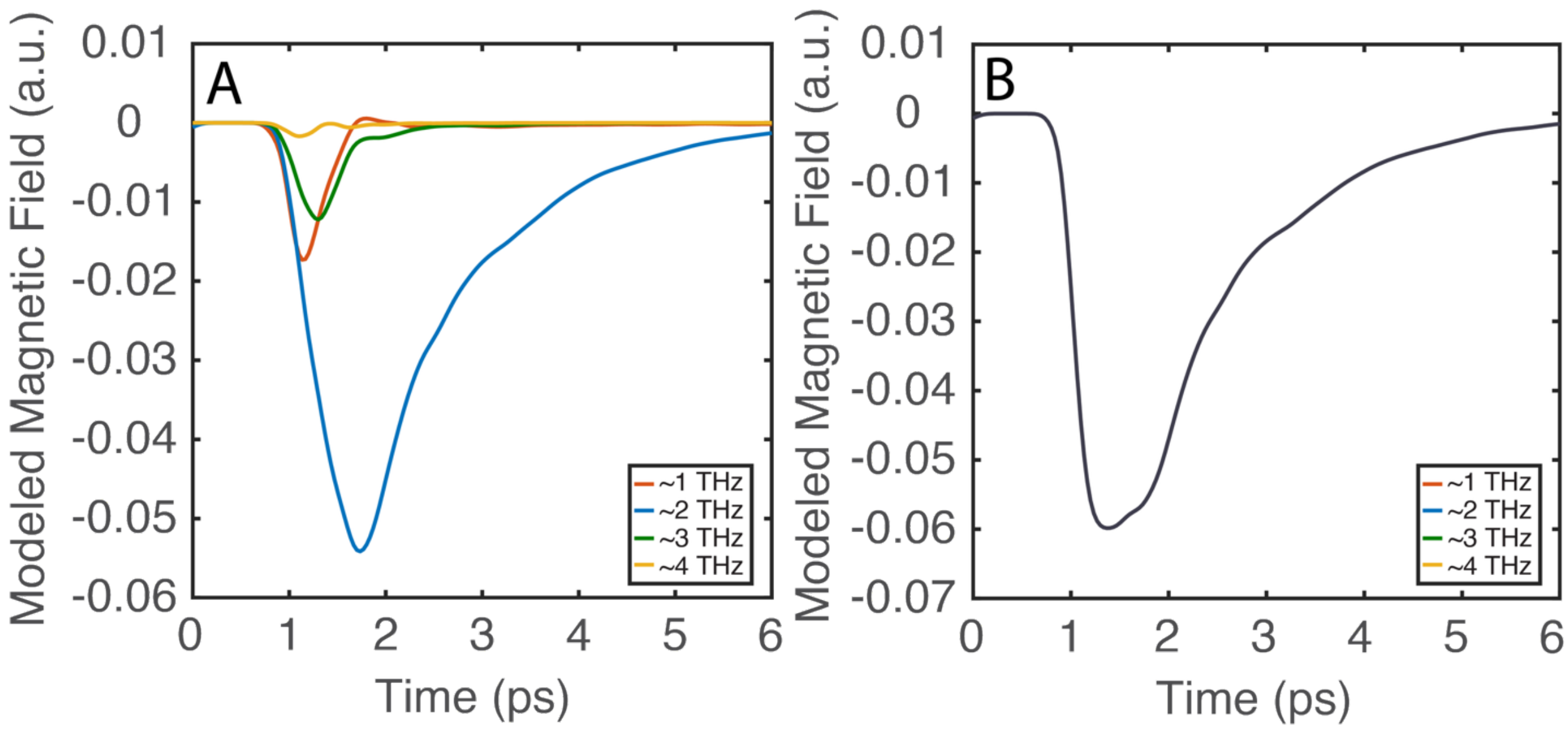

Fig. S5. **Modeled magnetic field.** (**A**) Modeled magnetic field from vibrational motion of the $E(TO_1)$ phonon-polariton at 4 frequencies. (**B**) Total modeled magnetic field from summing the magnetic field models for 4 frequencies.

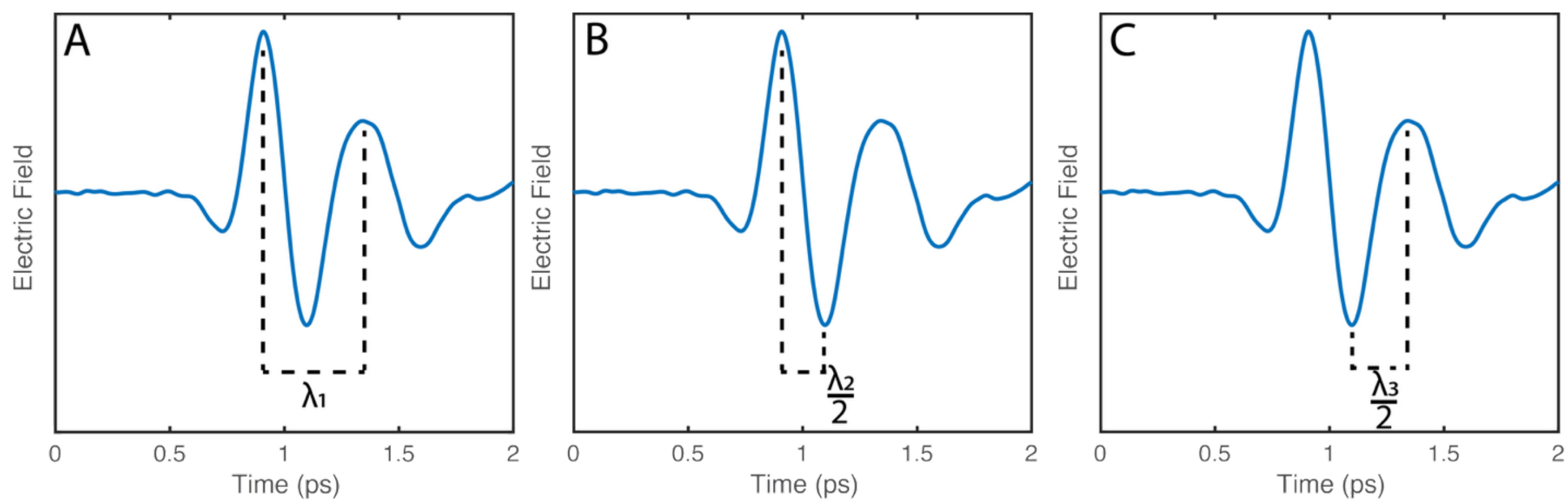

Fig. S6. Calculating the wavelength of the THz electric field strength in three ways. (**A**) Method 1: The distance between the first two minima. (**B**) Method 2: Twice the distance between the first maxima and the first minima. (**C**) Method 3: Twice the distance between the first minima and the second maxima.

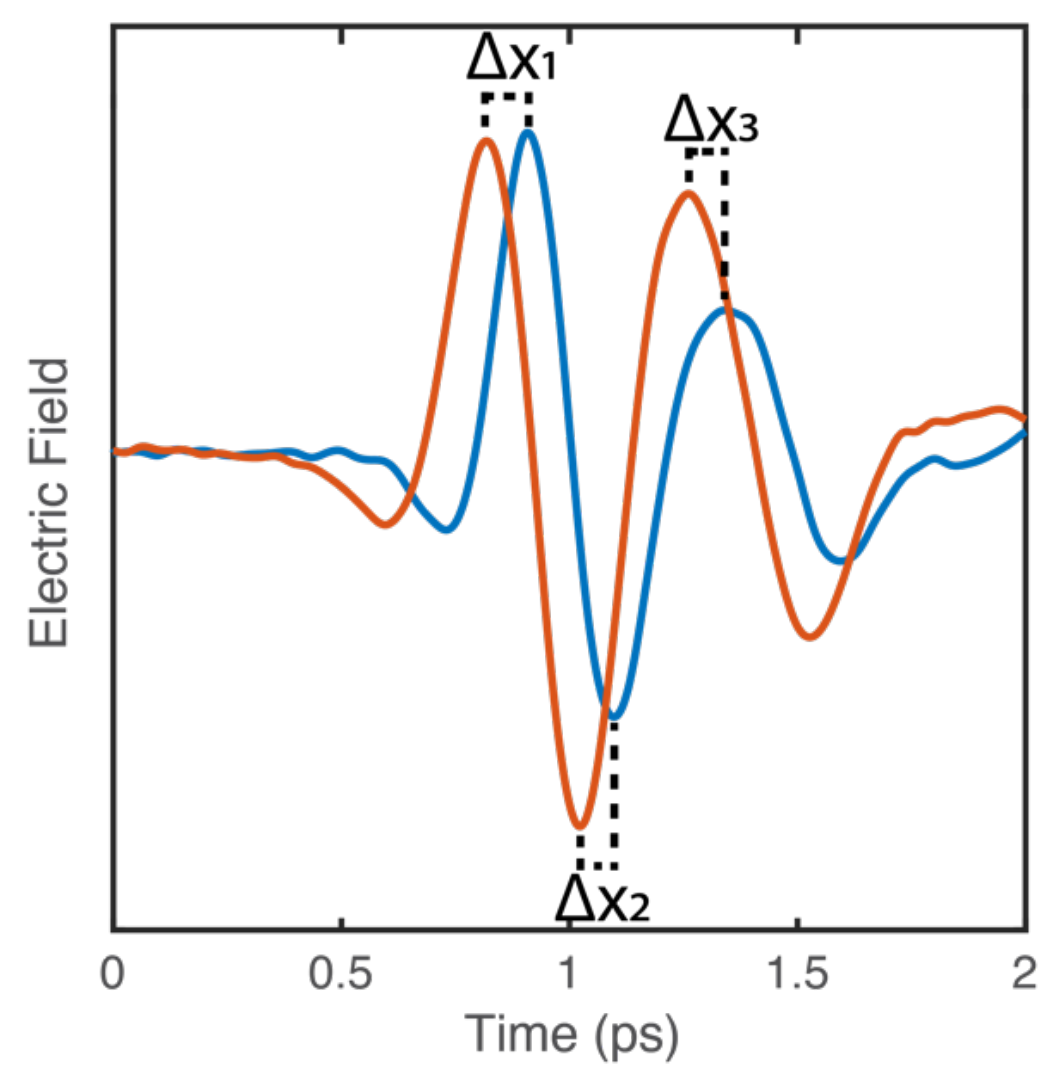

Fig. S7. Illustration of how the individual Δ*x*s are calculated.

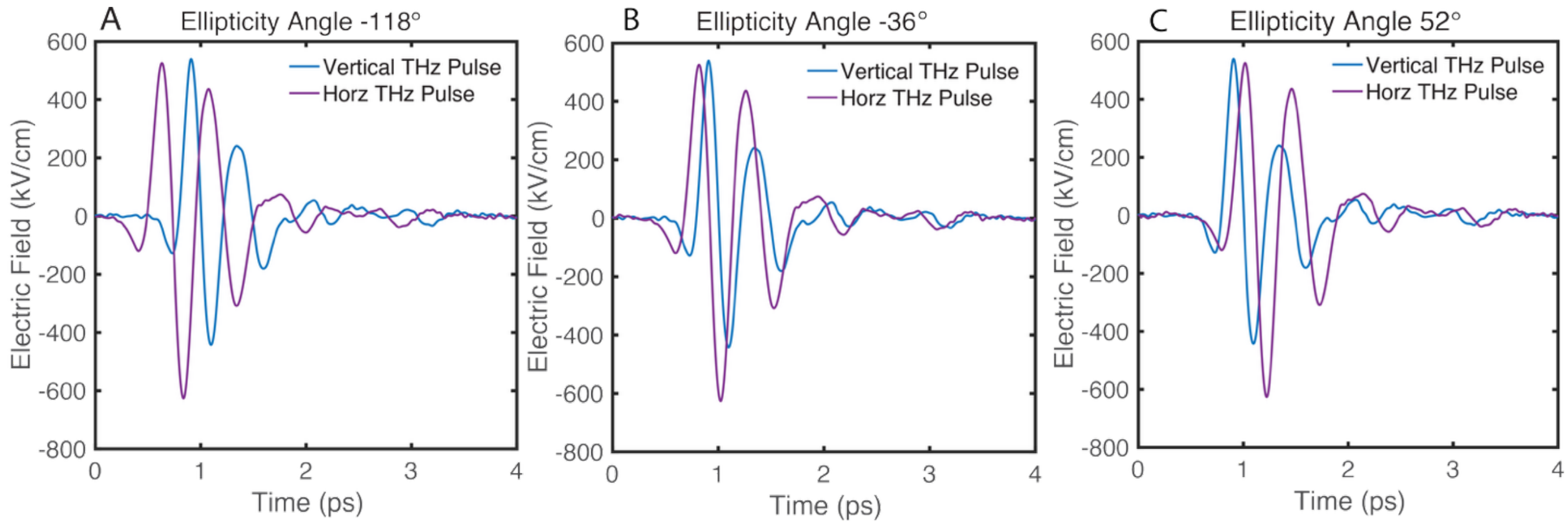

Fig. S8. Vertical and horizontal THz pulses at various ellipticity angles. Phase delay between the THz pulses at (**A**) -118 degrees ellipticity (**B**) -36 degrees ellipticity and (**C**) +52 degrees ellipticity.

REFERENCES

1. J. R. Barker, The magnetic field inside a Solenoid. *Br. J. Appl. Phys.* **1**, 65–67 (1950).

2. P. S. Pershan, J. P. van der Ziel, L. D. Malmstrom, Theoretical discussion of the inverse Faraday effect, raman scattering, and related phenomena. *Phys. Rev.* **143**, 574–583 (1966).

3. J. P. van der Ziel, P. S. Pershan, L. D. Malmstrom, Optically-induced magnetization resulting from the inverse Faraday effect. *Phys. Rev. Lett.* **15**, 190–193 (1965).

4. V. Plastovets, A. I. Buzdin, Fluctuation-mediated inverse Faraday effect in superconducting rings. *Phys. Lett. A* **481,** 129001 (2023).

5. O. H. Cheng, D. H. Son, M. Sheldon, Light-induced magnetism in plasmonic gold nanoparticles. *Nat. Photonics* **14**, 365–368 (2020).

6. A. Kirilyuk, A. V. Kimel, T. Rasing, Ultrafast optical manipulation of magnetic order. *Rev. Mod. Phys.* **82**, 2731–2784 (2010).

7. D. M. Juraschek, M. Fechner, A. V. Balatsky, N. A. Spaldin, Dynamical multiferroicity. *Phys. Rev. Mater.* **1**, 014401 (2017).

8. D. M. Juraschek, T. Meuman, P. Narang, Giant effective magnetic fields from optically driven chiral phonons in 4f paramagnets. *Phys. Rev. Res.* **4**, 013129 (2022).

9. D. M. Juraschek, N. A. Spaldin, Orbital magnetic moments of phonons. *Phys. Rev. Mater.* **3**, 064405 (2019).

10. J. Luo, T. Lin, J. Zhang, X. Chen, E. R. Blackert, R. Xu, B. I. Yakobson, H. Zhu, Large effective magnetic fields from chiral phonons in rare-earth halides. *Science* **382**, 698–702 (2023).

11. M. Basini, M. Pancaldi, B. Wehinger, M. Udina, V. Unikandanunni, T. Tadano, M. C. Hoffmann, A. V. Balatsky, S. Bonetti, Terahertz electric-field-driven dynamical multiferroicity in $SrTiO_3$. *Nature* **628**, 534–539 (2024).

12. D. M. Juraschek, R. M. Geilhufe, H. Zhu, M. Basini, P. Baum, A. Baydin, S. Chaudhary, M. Fechner, B. Flebus, G. Grissonnanche, A. I. Kirilyuk, M. Lemeshko, S. F. Maehrlein, M. Mignolet, S. Murakami, Q. Niu, U. Nowak, C. P. Romao, H. Rostami, T. Satoh, N. A. Spaldin, H. Ueda, L Zhang, Chiral Phonons. *Nat. Phys.* **21**, 1532–1540 (2025).

13. T. Zhang, S. Murakami, Chiral phonons entangled with multiple Hall effects and unified convention for pseudoangular momentum in two-dimensional materials. *Phys. Rev. B* **105**, 235204 (2022).

14. H. Zhu, J. Yi, M. Li, J. Xiao, L. Zhang, C. Yang, R. Kaindl, L. Li, Y. Wang, X. Zhang, Observation of chiral phonons. *Science* **359**, 579–582 (2018).

15. H. Ueda, M. García-Fernández, S. Agrestini, C. P. Romao, J. van den Brink, N. A. Spaldin, K. Zhou, U. Staub, Chiral phonons in quartz probed by X-rays. *Nature* **618**, 946–950 (2023).

16. Y. Gao, Y. Pan, J. Zhou, L. Zhang, Chiral phonon mediated high-temperature superconductivity. *Phys. Rev. B* **108**, 064510 (2023).

17. K. Kim, E. Vetter, L. Yan, C. Yang, Z. Wang, R. Sun, Y. Yang, A. H. Comstock, X. Li, J. Zhou, L. Zhang, W. You, D. Sun, J. Liu, Chiral-phonon-activated spin Seebeck effect. *Nat. Mater.* **22**, 322–328 (2023).

18. T. Zhang, S. Murakami, H. Miao, Weyl phonons: The connection of topology and chirality. *Nat. Commun.* **16**, 3560 (2025).

19. J. Fransson, Y. Kapon, L. Brann, S. Yochelis, D. D. Sasselov, Y. Paltiel, S. F. Ozturk, Chiral Phonons Enhance Ferromagnetism. *J. Phys. Chem. Lett.* **16**, 2001–2007 (2025).

20. X. Gong, M. Kargarian, A. Stern, D. Yue, H. Zhou, Z. Jin, V. M. Galitski, V. M. Yakovenko, J. Xia, Time-reversal symmetry-breaking superconductivity in epitaxial bismuth/nickel bilayers. *Sci. Adv.* **3**, e1602579 (2017).

21. X. Xiao, F. Zhan, W. Kong, J. Fan, R. Wang, X. Wu, Time-reversal symmetry broken quantum spin Hall phase in the van der Waals heterostructure $ZrTe_5/Cr_2Ge_2Te_6$. *New J. Phys.* **24**, 093029 (2022).

22. G. de Vecchi, G. Jotzu, M. Buzzi, S. Fava, T. Gebert, M. Fechner, A. V. Kimel, A. Cavalleri, Generation of ultrafast magnetic steps for coherent control. *Nat. Photonics* **19**, 601–606 (2025).

23. D. M. Juraschek, P. Narang, N. A. Spaldin, Phono-magnetic analogs to opto-magnetic effects. *Phys. Rev. Res.* **2**, 043035 (2020).

24. R. Merlin, Unraveling the effect of circularly polarized light on reciprocal media: Breaking time reversal symmetry with non-Maxwellian magnetic-esque fields. arXiv:2309.13622 [physics.optics] (2023).

25. B. E. Knighton, B. S. Dastrup, C. L. Johnson, J. A. Johnson, Measurement of a phonon-polariton dispersion curve by varying the excitation wavelength. *Phys. Rev. B* **97**, 214307 (2018).

26. H. Lin, G. Mead, G. A. Blake, Mapping $LiNbO_3$ phonon-polariton nonlinearities with 2D THz-THz-Raman spectroscopy. *Phys. Rev. Lett.* **129**, 207401 (2022).

27. Y. Ikegaya, H. Sakaibara, Y. Minami, I. Katayama, J. Takeda, Real-time observation of phonon-polariton dynamics in ferroelectric $LiNbO_3$ in time-frequency space. *Appl. Phys. Lett.* **107**, 062901 (2015).

28. A. Schneider, M. Neis, M. Stillhart, B. Ruiz, R. U. A. Khan, P. Günter, Generation of terahertz pulses through optical rectification in organic DAST crystals: Theory and experiment. *J. Opt. Soc. Am. B* **23**, 1822–1835 (2006).

29. C. Rader, M. F. Nielson, B. E. Knighton, Z. B. Zaccardi, D. J. Michaelis, J. A. Johnson, Custom terahertz waveforms using complementary organic nonlinear optical crystals. *Opt. Lett.* **47**, 5985–5988 (2022).

30. B. S. Dastrup, J. R. Hall, J. A. Johnson, Experimental determination of the interatomic potential in $LiNbO_3$ via ultrafast lattice control. *Appl. Phys. Lett.* **110**, 162901 (2017).

31. B. E. Knighton, R. T. Hardy, C. L. Johnson, L. M. Rawlings, J. T. Woolley, C. Calderon, A. Urrea, J. A. Johnson, Terahertz waveform considerations for nonlinearly driving lattice vibrations. *J. Appl. Phys.* **125**, 144101 (2019).

32. M. F. Biggs, B. E. Knighton, A. Alejandro, L. M. Davis, C. Rader, J. A. Johnson, Pump Pulse Bandwidth-Activated Nonlinear Phononic Coupling in CdWO4. arXiv:2310.08747 [physics.optics] (2023).

33. J. Lu, X. Li, Y. Zhang, H. Y. Hwang, B. K. Ofori-Okai, K. A. Nelson, Two-Dimensional Spectroscopy at Terahertz Frequencies. *Top. Curr. Chem.* **376**, 6 (2018).

34. A. Liu, A. Disa, Excitation-dependent features and artifacts in 2-D terahertz spectroscopy. *Opt. Express* **32**, 28160 (2024).

35. K. A. Grishunin, V. R. Bilyk, E. D. Mishina, A. V. Kimel, E. A. Mashkovich, Two-dimensional terahertz spectroscopy as a tool for revealing nonlinear interactions in media. *Rev. Sci. Instrum.* **94**, 073005 (2023).

36. C. L. Johnson, B. E. Knighton, J. A. Johnson, Distinguishing Nonlinear Terahertz Excitation Pathways with Two-Dimensional Spectroscopy. *Phys. Rev. Lett.* **122**, 073901 (2019).

37. T. G. H. Blank, K. A. Grishunin, K. A. Zvezdin, N. T. Hai, J. C. Wu, S. -H. Su, J. -C. A. Huang, A. K. Zvezdin, A. V. Kimel, Two-Dimensional Terahertz Spectroscopy of Nonlinear Phononics in the Topological Insulator $MnBi_2Te_4$. *Phys. Rev. Lett.* **131**, 026902 (2023).

38. S. Kase, K. Ohi, Optical-absorption and interband Faraday-rotation in $LiTaO_3$ and $LiNbO_3$. *Ferroelectrics.* **8**, 419–420 (1974).

39. J. Shan, J. I. Dadap, T. F. Heinz, Circularly polarized light in the single-cycle limit: The nature of highly polychromatic radiation of defined polarization. *Opt. Express* **17**, 7431–7439 (2009).

40. C. Rader, Z. B. Zaccardi, S. E. Ho, K. G. Harrell, P. K. Petersen, M. F. Nielson, H. Stephan, N. K. Green, D. J. H. Ludlow, M. J. Lutz, S. J. Smith, D. J. Michaelis, J. A. Johnson, A new

standard in high-field terahertz generation: The organic nonlinear optical crystal PNPA. *ACS Photonics* **9**, 3720–3726 (2022).
